# Dryland soil chemical properties and crop yields affected by long-term tillage and cropping sequence

**DOI:** 10.1186/s40064-015-1122-4

**Published:** 2015-07-05

**Authors:** Upendra M Sainju, Brett L Allen, Thecan Caesar-TonThat, Andrew W Lenssen

**Affiliations:** USDA-ARS, Northern Plains Agricultural Research Laboratory, Sidney, MT 59270 USA; Department of Agronomy, Iowa State University, Ames, IA 50011 USA

**Keywords:** Chemical properties, Crop yields, Nutrients, Tillage, Crop rotation

## Abstract

Information on the effect of long-term management on soil nutrients and chemical properties is scanty. We examined the 30-year effect of tillage frequency and cropping sequence combination on dryland soil Olsen-P, K, Ca, Mg, Na, SO_4_–S, and Zn concentrations, pH, electrical conductivity (EC), and cation exchange capacity (CEC) at the 0–120 cm depth and annualized crop yield in the northern Great Plains, USA. Treatments were no-till continuous spring wheat (*Triticum aestivum* L.) (NTCW), spring till continuous spring wheat (STCW), fall and spring till continuous spring wheat (FSTCW), fall and spring till spring wheat–barley (*Hordeum vulgare* L., 1984–1999) followed by spring wheat–pea (*Pisum sativum* L., 2000–2013) (FSTW-B/P), and spring till spring wheat-fallow (STW-F, traditional system). At 0–7.5 cm, P, K, Zn, Na, and CEC were 23–60% were greater, but pH, buffer pH, and Ca were 6–31% lower in NTCW, STCW, and FSTW–B/P than STW-F. At 7.5–15 cm, K was 23–52% greater, but pH, buffer pH, and Mg were 3–21% lower in NTCW, STCW, FSTCW, FSTW–B/P than STW-F. At 60–120 cm, soil chemical properties varied with treatments. Annualized crop yield was 23–30% lower in STW-F than the other treatments. Continuous N fertilization probably reduced soil pH, Ca, and Mg, but greater crop residue returned to the soil increased P, K, Na, Zn, and CEC in NTCW and STCW compared to STW-F. Reduced tillage with continuous cropping may be adopted for maintaining long-term soil fertility and crop yields compared with the traditional system.

## Background

Long-term sustainability of dryland farming systems depends on soil quality and fertility (Karlen et al. 1997; Liebig et al. 2002). Poor soil management practices can lead to degraded soil and environmental quality and reduction in crop yields (Karlen et al. 1997). Novel management techniques are needed to maintain the long-term sustainability of soil resources and crop yields without seriously degrading the environment (Lal et al. 1994; Karlen et al. 1997; Liebig et al 2002).

In the northern Great Plains, wheat-fallow systems have been used as the traditional dryland farming practice since the last century (Peterson et al 1998; Halvorson et al. 2000, 2002). In these systems, land is typically fallowed from 14 to 20 mo. Fallowing is used to conserve soil water, release plant nutrients, control weeds, increase succeeding crop yields, and reduce the risk of crop failure (Aase and Pikul 1995; Jones and Popham 1997). Fallowing, however, can reduce soil quality and fertility by increasing organic matter mineralization and erosion and decrease crop yields by the absence of crop during the fallow period (Aase and Pikul 1995; Halvorson et al. 2000, 2002; Sainju et al. 2007, 2009). As a result, the traditional farming system has become inefficient, uneconomical, and unsustainable (Aase and Schaefer 1996).

Alternate-year fallowing and crop rotation can also affect soil nutrients and chemical properties compared with continuous monocropping. Sainju et al. (2011) found that Mg content was lower at the subsurface layer, but SO_4_–S content was higher in the surface layer in wheat-fallow than continuous wheat after 9 year in dryland cropping systems in western Montana. Lal et al. (1994) reported that Ca and Mg contents and CEC were greater in legume-nonlegume rotation than continuous nonlegume after 28 year in Ohio. The amount of nutrients removed through grain harvest can be higher in continuous cropping than crop-fallow due to increased annualized yield, thereby reducing soil nutrient contents under the continuous cropping system (Sainju et al. 2009; Sainju 2013). Continuous application of NH_4_-based N fertilizers to nonlegume crops can reduce soil pH compared with legume-nonlegume crop rotation where N fertilizer is not applied to legumes (Lal et al. 1994; Liebig et al. 2002). After 16–28 year of management implications, soil pH was reduced by 0.22–0.42 in continuous nonlegumes compared with crop rotation containing legumes and nonlegumes (Lal et al. 1994; Liebig et al. 2002). Soil acidification from N fertilization to crops primarily results from (1) increased removal of basic cations, such as Ca, Mg, K, and Na in crop grains and stover due to increased yield, (2) leaching of soil residual NO_3_-N, Ca, and Mg, and (3) microbial oxidation (or nitrification) of NH_4_-based N fertilizers that release H^+^ ions (Mahler and Harder 1984). Alkalinity produced during plant uptake of N or conversion of inorganic N to organic form, however, can partly or wholly counter the acidity from nitrification (Schroder et al. 2011). Increased toxicity of Al and Mn and reduced availability of most nutrients, such as Ca, Mg, K, and Na, during acidification can reduce crop growth and yield (Tumuslime et al. 2011).

Tillage also affects soil chemical properties due to differences in residue placement in the soil and removal of nutrients in grains as a result of variations in crop yields. Tarkalson et al. (2006) observed that no-tillage increased soil Bray-P and CEC, but reduced K, Ca, base saturation, and pH compared with conventional tillage at 0–5 cm after 27 year under dryland spring wheat–sorghum (*Sorghum vulgare* L.)/corn (*Zea mays* L.)-fallow in Nebraska. The trend reversed at 5–10 cm. They reported that placement of residue at the soil surface and increased nutrient removal due to higher crop yields influenced nutrient levels and chemical properties in no-tillage compared with residue incorporation to a greater depth in conventional tillage. Similarly, Lal et al. (1994) found greater CEC at 0–15 cm in no-tillage than conventional tillage after 28 year in Ohio. Sainju et al. (2011) reported lower soil pH, Ca, and Na contents at 0–30 cm in no-tillage than conventional tillage after 9 year in western Montana. Nitrogen fertilizers are usually placed at the soil surface and N rates are higher for no-tillage due to accumulation of surface residue that partly immobilize N than conventional tillage where fertilizers are incorporated into the soil due to tillage (Zibilski et al. 2002). The surface application of N fertilizers also reduces soil pH in no-tillage more than conventional tillage (Lilienfein et al. 2000).

Little is known about the long-term (30 year) impact of tillage and cropping sequence combination on soil nutrients and chemical properties in dryland cropping systems in the northern Great Plains, USA. Our objectives were to: (1) evaluate the 30-year influence of tillage and cropping sequence combination on dryland annualized crop yield and soil Olsen-P, K, Ca, Mg, Na, SO_4_–S, and Zn concentrations and pH, buffer pH, CEC, and EC at the 0–120 cm depth under dryland cropping systems in eastern Montana, USA and (2) determine a management practice that can enhance long-term sustainability of soil fertility and crop yields. The study included different combination of tillage frequency (no-tillage, spring tillage, and spring and fall tillage) and crop rotations (with spring wheat, barley, pea, and fallow in the rotation) from 1984 to 2013 as described below. We hypothesized that reduced tillage with continuous cropping would increase annualized crop yields and improve soil nutrients and chemical properties compared with the traditional system of conventional tillage with spring wheat-fallow.

## Methods

### Field experiment

The experiment was initiated by Aase and Pikul (1995) in 1983 at a dryland farm site 11 km north of Culbertson (48° 33′N, 104° 50′W), eastern Montana, USA. The site is characterized by wide variation in mean monthly air temperature ranging from −8°C in January to 23°C in July and August and a mean (68-year average) annual precipitation of 340 mm, 70% of which occurs during the growing season (April–August). The soil is a Dooley sandy loam (fine loamy, mixed, frigid, Typic Argiboroll) with 2–6% slope. The soil sampled in 1983 prior to the initiation of the experiment had 645 g kg^−1^ sand, 185 g kg^−1^ silt, 170 g kg^−1^ clay, 1.50 Mg m^−3^ bulk density, 14.9 g C kg^−1^ soil organic C, and 6.2 pH at the 0–7.5 cm depth (Aase and Pikul 1995). The pH, however, increased to 8.7 at 90–120 cm.

Details of the experimental treatments and management practices conducted from 1983 to 2013 are shown in Table 1. In brief, the treatments consisted of no-till continuous spring wheat (NTCW), spring till continuous spring wheat (STCW), fall and spring till continuous spring wheat (FSTCW), fall and spring till spring wheat–barley (1984–1999) followed by spring wheat–pea (2000–2013) (FSTW-B/P), and spring till spring wheat-fallow (STW-F). The cropping sequences were continuous spring wheat in NTCW, STCW, and FSTCW and two-year rotations of spring wheat–barley followed by spring wheat–pea in FSTW–B/P and spring wheat-fallow in STW-F. Each phase of the crop rotation was present in every year. In STCW, plots were tilled with a field cultivator with 0.45 m wide medium crown prior to spring wheat seeding to prepare a seedbed in the spring. In FSTCW and FSTW-B/P, plots were tilled with a field cultivator in the fall, followed by tandem disk tillage in the spring to prepare the seedbed. Similarly, in STW-F, plots were tilled with tandem disk prior to seeding in the spring. Tillage was started from fall 1983 in FSTCW and FSTW–B/P and from spring 1984 in STCW and STW-F. Treatments were arranged in a randomized complete block with four replications. Individual plot size was 12 m × 30 m.

**Table 1:** Crop management used for various treatments from 1983 to 2013 at the experimental site

Management	NTCW	STCW	FSTCW	FSTW-B/P	STW-F
Crops	Continuous spring wheat	Continuous spring wheat	Continuous spring wheat	Spring wheat–barley (1984–1999), spring wheat–pea (2000–2013)	Spring wheat-fallow
Tillage	No-tillage	Field cultivator in April	Field cultivator in April, tandem disk in September	Field cultivator in April, tandem disk in September	Field cultivator in April, tandem disk in September, and as needed in other times
Planting	Late April–early May	Late April–early May	Late April–early May	Late April–early May	Late April–early May
N fertilizer	56 kg N ha^−1^ (1984–1985) and 34 kg N ha^−1^ (1986–1996) as NH_4_NO3, 70 kg N ha^−1^ (1997–2013) as urea and monoammonium phosphate (MAP)	56 kg N ha^−1^ (1984–1985) and 34 kg N ha^−1^ (1986–1996) as NH_4_NO3, 70 kg N ha^−1^ (1997–2013) as urea and MAP	56 kg N ha^−1^ (1984–1985) and 34 kg N ha^−1^ (1986–1996) as NH_4_NO3, 70 kg N ha^−1^ (1997–2013) as urea and MAP	Wheat: 56 kg N ha^−1^ (1984–1985) and 34 kg N ha^−1^ (1986–1996) as NH_4_NO3, 70 kg N ha^−1^ (1997–2013) as urea and MAP Barley: 56 kg N ha^−1^ (1984–1985) and 34 kg N ha^−1^ (1986–1996) as NH_4_NO3, 70 kg N ha^−1^ (1997–1999) as urea and MAP Pea: 5 kg N ha^−1^ as MAP (2000–2013)	34 kg N ha^−1^ (1984–1996) as NH_4_NO3, 70 kg N ha^−1^ (1997–2013) as urea and MAP to wheat
P fertilizer	46 kg P ha^−1^ (1984–1996), 29 kg P ha^−1^ (1997–2013)	46 kg P ha^−1^ (1984–1996), 29 kg P ha^−1^ (1997–2013)	46 kg P ha^−1^ (1984–1996), 29 kg P ha^−1^ (1997–2013)	46 kg P ha^−1^ (1984–1996), 29 kg P ha^−1^ (1997–2013) to all crops	46 kg P ha^−1^ (1984–1996), 29 kg P ha^−1^ (1997–2013) to wheat
K fertilizer	No K fertilizer (1984–1996); 48 kg K ha^−1^ (1997–2013)	No K fertilizer (1984–1996); 48 kg K ha^−1^ (1997–2013)	No K fertilizer (1984–1996); 48 kg K ha^−1^ (1997–2013)	No K fertilizer (1984–1996); 48 kg K ha^−1^ (1997–2013) to all crops	No K fertilizer (1984–1996); 48 kg K ha^−1^ (1997–2013) to wheat
Varieties and seed rate	Lew (1983–1996), McNeal (1997–2005), Reeder (2006–2009), Vida (2010–2013) at 74 kg ha^−1^	Lew (1983–1996), McNeal (1997–2005), Reeder (2006–2009), Vida (2010–2013) at 74 kg ha^−1^	Lew (1983–1996), McNeal (1997–2005), Reeder (2006–2009), Vida (2010–2013) at 74 kg ha^−1^	Wheat: Lew (1983–1996), McNeal (1997–1999) at 74 kg ha^−1^, Barley: Certified tradition (1984–1999) at 84 kg ha^−1^, Pea: Majoret (2000–2009) and Cruiser (2010–2013) at 160 kg ha^−1^	Lew (1983–1996), McNeal (1997–2005), Reeder (2006–2009), Vida (2010–2013) at 74 kg ha^−1^
Weed control	Herbicide	Herbicide and tillage	Herbicide and tillage	Herbicide and tillage	Herbicide and tillage
Harvest	Grain: Bundle sample (5 m^2^, 1984–1995), Swathe (15–45 m^2^, 1996–2013) in July–August. Biomass 1 m^2^ in July–August	Grain: Bundle sample (5 m^2^, 1984–1995), Swathe (15–45 m^2^, 1996–2013) in July–August. Biomass 1 m^2^ in July–August	Grain: Bundle sample (5 m^2^, 1984–1995), Swathe (15–45 m^2^, 1996–2013) in July–August. Biomass 1 m^2^ in July–August	Grain: Bundle sample (5 m^2^, 1984–1995), Swathe (15–45 m^2^, 1996–2013) in July–August. Biomass 1 m^2^ in July–August	Grain: Bundle sample (5 m^2^, 1984–1995), Swathe (15–45 m^2^, 1996–2013) in July–August. Biomass 1 m^2^ in July–August

The rate and source of N, P, and K fertilizers, spring wheat, barley, and pea varieties, and seeding rates for each treatment are shown in Table 1. Fertilizers were broadcast at planting in April–May, 1984–2013, left above the soil surface in the no-till system and incorporated to a depth of 10 cm using tillage in the till systems. Wheat, barley, and pea were planted using a no-till drill at row spacing of 20 cm. Pea seeds were inoculated with proper *Rhizobium sp.* before planting. Biomass sample was harvested 2 days before grain harvest in August. Both biomass (stems + leaves) and grain samples were oven-dried at 65°C for yield determination. Crop biomass residue was returned to the soil after harvesting grains from the rest of the area in each plot.

### Soil sample collection and analysis

In October 2013, soil samples were collected with a truck-mounted hydraulic probe (3.5 cm inside diameter) from the 0–120 cm depth from five places in the central rows of each plot, separated into 0–7.5, 7.5–15, 15–30, 30–60, 60–90, and 90–120 cm depths, and composited within a depth. Samples were air-dried, ground, and sieved to 2 mm for determining nutrient concentrations and chemical properties.

Soil samples were analyzed for Olsen-P, K, Ca, Mg, Na, SO_4_–S, and Zn concentrations, and CEC, EC, pH, and buffer pH in Agvise Laboratories, Northwood, ND. Olsen-P was determined by extracting the soil with buffered alkaline solution (NaHCO_3_–NaOH) and determining P concentration in the solution using a colorimeter (Kuo 1996). Concentrations of K, Ca, Mg, and Na were determined using an atomic absorption and flame emission spectrometry after extracting the soil with NH_4_OAc solution (Wright and Stuczynski 1996). Sulfate-S was determined by the methylene blue method (Tabatabai 1996). Soil pH was determined with a pH meter in 1:2 soil/water solution and buffer pH in 1:2 soil/0.5 mol L^−1^ KCl solution. The CEC was determined by the method described by Sumner and Miller (1996) for arid region soils. The EC was determined with a conductance meter in 1:1 soil/water paste (Rhoades 1996).

As P and K fertilizers were applied to crops from 1984 to 2013, P and K balances were calculated for each treatment using soil total P and K contents at the 0–120 cm depth at the beginning (1983) and end (2013) of the experiment using the following equation:

P or K balance = Final soil total P or K contents in 2013 (0–120 cm) + Total P or K removed in crops grains from 1984 to 2013 − Initial soil total P or K contents in 1983 (0–120 cm) − Total amount of P or K fertilizers applied to crops from 2013 to 2014.

For calculating P and K balances, values for soil total P content at the beginning and end of the experiment and K content at the beginning of the experiment were either not known or measured. As a result, total P content (kg P ha^−1^) at 0–120 cm at the beginning and end of the experiment was calculated by dividing soil organic C (SOC, kg C ha^−1^) by the estimated SOC/total P ratio of 58 for cultivated soils (Kirkby et al. 2011). Similarly, K content at 0–120 cm at the beginning of the experiment was calculated by dividing SOC by the estimated SOC/total K ratio of 30 for cultivated soils (Wang et al. 2014). The SOC content (kg C ha^−1^) at 0–120 cm at the beginning of the experiment (1983) as estimated from a nearby grassland soil was 134,700 kg C ha^−1^. At the end of the experiment (2013), SOC contents at 0-120 cm were 130,900, 122,100, 126,700, 122,300, and 118,400 kg C ha^−1^ for NTCW, STCW, FSTCW, FSYW-B/P, and STW-F, respectively (unpublished data). Total P and K removed in crop grains were determined by multiplying mean annualized crop yield by the estimated P concentration of 2.2 g P kg^−1^ and K concentration of 2.5 g K kg^−1^ for spring wheat, barley, and pea grain yield (Murdock et al. 2009) and by 30 (total number of years). Nitrogen balance for this experiment had been discussed in a separate paper.

### Data analysis

Data for annualized crop yield from 1984 to 2013 and soil parameters in 2013 were analyzed using the SAS-MIXED model (Littell et al. 1996). For crop yield, treatment and treatment × year interaction were considered as fixed effects, year as the repeated measure variable, and replication as the random effect. For soil parameters, treatment and treatment × soil depth interaction were considered as fixed effects, depth as the repeated measure variable, and replication as the random effect. Because each phase of the cropping sequence in two-year rotations was present in every year, data for phases were averaged within a sequence and the averaged value was used for the analysis. As crop was absent during the fallow phase of the sequence, yield of spring wheat during the crop year in STW-F rotation was divided by 2 to calculate the annualized yield. In FSTW-B/P, annualized yield was calculated by averaging the yield of spring wheat and barley or pea. Means were separated by using the least square means test when treatments and interactions were significant (Littell et al. 1996). Statistical significance was evaluated at *P* ≤ 0.05, unless otherwise stated.

## Results and discussion

### Annualized crop yield

**Figure 1:**
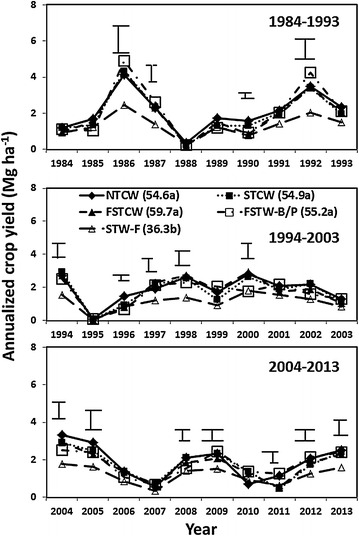
Effect of tillage and cropping sequence combination on annualized crop yields from 1984 to 2013. *FSTCW* denotes fall and spring till continuous spring wheat, *FSTW–B/P* fall and spring till spring wheat–barley (1984–1999) followed by spring wheat–pea (2000–2013), *NTCW* no-till continuous spring wheat, *STCW* spring till continuous spring wheat and *STW-F* spring till spring wheat-fallow. Annualized crop yield in FSTW–B/P includes average yield of spring wheat and barley from 1984 to 1999 and spring wheat and pea from 2000 to 2013. *Bars* at the *top* denote least significant difference among treatments at *P* = 0.05. *Number* in *parenthesis* along with *different letters* in the treatment legend denotes significant mean annualized crop yield from 1984 to 2013.

Annualized crop grain yield varied with treatments and years, with a significant (*P* ≤ 0.05) treatment × year interaction. Grain yield was greater in NTCW, STCW, FSTCW, and FSTW–B/P than STW-F in 1986, 1987, 1992, 1994, 1997, 1998, 2004, 2005, 2009, 2012, and 2013 (Figure 1). In 1990 and 1996, grain yield was greater in NTCW than FSTCW, FSTW-B/P, and STW-F. In 2000 and 2011, grain yield was greater in NTCW and FSTW–B/P than STW-F and STCW. In 2008, grain yield was greater in STCW than STW-F. Averaged across years, grain yield was 23 to 30% lower in STW-F than NTCW, STCW, FSTCW, and FSTW–B/P (Figure 1). Biomass (stems + leaves) yield also followed trends similar to grain yield.

**Figure 2:**
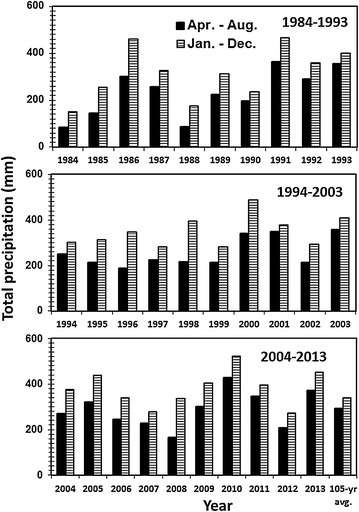
Total precipitation during the growing season (April–August) and throughout the year (January–December) from 1984 to 2013 at the experimental site.

Absence of crops during fallow resulted in lower annualized crop grain yield in STW-F than the other treatments during the years when the growing season precipitation was near or similar to the 105-year average (Figure 2). Similar results of lower annualized crop grain yield in crop-fallow than continuous cropping in dryland cropping systems during the years with near normal precipitation in the northern Great Plains have been reported by several researchers (Halvorson et al 2000; Campbell et al 2004; Tarkalson et al. 2006; Sainju 2014). Crop yields were not different among treatments during years with below-normal precipitation, such as in 1984, 1985, 1988, 1995, 2006, and 2007 or above-average precipitation, such as in 1991, 1993, 2003, and 2010. This suggests that increased soil water conservation by fallow during years with below-normal precipitation increased crop yield in STW-F, thereby resulting in similar annual crop yields among all treatments in these years. During years with above-average precipitation, anaerobic condition due to increased soil water content reduced crop yields, resulting in non-significant differences in yields among all treatments.

Orthogonal contrasts indicated non-significant differences among NTCW, STCW, and FSTCW, suggesting that tillage had no effect on crop yield. Several researchers (Halvorson et al 2000; Sainju et al. 2009; Lenssen et al 2014) also found that tillage had minimal effect on dryland crop grain yields. Similarly, non-significant difference in yields between FSTCW and FSTW–B/P indicates that crop rotation also had no effect on annualized grain yield compared with monocropping. Greater average yields in NTCW, STCW, FSTCW, and FSTW–B/P than STW-F suggest that continuous cropping can increase annualized crop yield compared with crop-fallow under dryland cropping systems in the northern Great Plains. Differences in grain yields, nutrient removal in grain, and the amount of crop residue returned to the soil resulted in variations in soil chemical properties and nutrient concentrations, as described below.

### Soil phosphorus and potassium

Soil Olsen-P concentration varied among treatments and soil depths, with a significant treatment × depth interaction (Table 2). At 0–7.5 cm, Olsen-P was greater in NTCW, STCW, FSTCW, and FSTW–B/P than STW-F (Table 3). At other depths, treatment had no effect on Olsen-P and averaged 4.6, 2.4, 2.0, 2.1, and 2.8 mg P kg^−1^ at 7.5–15, 15–30, 30–60, 60–90, and 90–120 cm, respectively. Olsen-P was greater at 0–7.5 cm but lower at 15–30 cm under continuous wheat than wheat-fallow. Similarly, Olsen P at 7.5–15 cm was lower under continuous wheat than wheat–barley/pea. Olsen-P concentration decreased from 0–7.5 to 7.5–15 cm and remained constant thereafter at other depths in all treatments. After 30 year, P balance at 0–120 cm was greater in STW-F than STCW and FSTW–B/P (Table 4). Phosphorus balance was lower under continuous wheat than wheat-fallow, but greater than wheat–barley/pea.

**Table 2:** Analysis of variance for the effects of tillage and cropping sequence combination treatment and soil depth on soil chemical properties

Parameter	Treatment (T)	Soil depth (D)	T × D
Olsen-P	**	***	***
K	***	***	*
Ca	NS^a^	***	*
Mg	NS	***	*
Na	***	***	*
SO_4_–S	**	*	*
Zn	NS	***	*
Electrical conductivity (EC)	NS	**	*
Cation exchange capacity (CEC)	**	**	*
pH	*	**	*
Buffer pH	*	**	*

* Significant at *P* ≤ 0.05.

** Significant at *P* ≤ 0.01.

*** Significant at *P* ≤ 0.001.

^a^Not significant.

**Table 3:** Effect of tillage and cropping sequence combination on soil Olsen-P, K, and SO_4_–S concentrations at the 0–120 cm depth in 2013

Tillage and cropping sequence^a^	Soil depth					
	0–7.5 cm	7.5–15 cm	15–30 cm	30–60 cm	60–90 cm	90–120 cm
Olsen-P concentration (mg P kg^−1^)						
NTCW	36.8a^b^A^c^	2.8B	2.3B	2.0B	2.3B	3.3B
STCW	40.0aA	5.5B	2.0C	2.0C	2.0C	2.0C
FSTCW	34.3aA	3.0B	2.3B	2.0B	2.1B	3.3B
FSTW-B/P	36.6aA	6.0B	2.1C	2.1C	1.9C	2.6C
STW-F	25.0bA	4.9B	3.3BC	2.0C	2.2B	2.6BC
Contrast						
NT vs. T	−0.4	−1.5	0.1	0.1	0.3	0.7
CW vs. W-F	15.0***	0.63	−1.3*	0.1	−0.2	−0.6
CW vs. W-B/P	−2.4	−3.6*	0.1	−0.1	0.2	0.7
K concentration (mg K kg^−1^)						
NTCW	331aA	279aB	157C	96D	91D	105D
STCW	331aA	228B	179B	112C	98C	96C
FSTCW	325abA	263aB	150C	97D	91D	100D
FSTW-B/P	348aA	242aB	149C	103C	102C	115C
STW-F	272bA	186bB	129C	93C	98C	95C
Contrast						
NT vs. T	3.0	6.5	−7.0	−8.9	−3.1	6.9
CW vs. W-F	58.1*	96.6**	49.6*	18.4*	0.1	1.0
CW vs. W-B/P	−23.9	21.5	0.4	−5.3	−11.5	−15.6
SO_4_–S concentration (mg SO_4_–S kg^−1^)						
NTCW	6.8A	3.5A	3.0A	3.0A	4.3A	5.0bA
STCW	6.3A	3.5A	3.0A	3.3A	4.8A	5.8bA
FSTCW	6.0B	3.5B	3.3B	4.3B	10.5AB	26.5aA
FSTW-B/P	12.8B	11.0B	11.8B	12.3AB	21.0A	23.9aA
STW-F	8.0A	8.1A	7.5A	10.4A	11.4A	11.8bA
Contrast						
NT vs. T	0.6	0.1	−0.1	−0.8	−3.4	−11.3*
CW vs. W-F	−1.8	−4.6	−4.5	−7.1	−6.6	−6.0
CW vs. W-B/P	−6.8	−7.5	−8.5	−8.0	−10.5	2.6

*NTCW* no-till continuous spring wheat, *STCW* spring till continuous spring wheat and *STW-F* spring till spring wheat-fallow. *CW* continuous wheat, *NT* no-till, *T* till, *W–B/P* spring wheat–barley/pea and *W-F* spring wheat-fallow.

*, **, and *** Significant at *P* = 0.05, 0.01, and 0.001, respectively.

^a^Tillage and cropping sequence are *FSTCW* fall and spring till continuous spring wheat, *FSTW–B/P* fall and spring till spring wheat–barley (1994–1999) followed by spring wheat–pea (2000–2013).

^b^Numbers followed by different lowercase letters within a column among treatments in a set are significantly different at *P* ≤ 0.05 by the least square means test.

^c^Numbers followed by different uppercase letters within a row among soil depths in a set are significantly different at *P* ≤ 0.05 by the least square means test.

**Table 4:** Effects of tillage and cropping sequence combination on P and K balance at 0–120 cm depth after 30 year (1983–2013)

Treatment^a^	Initial total content in the soil in 2013 (A)	Total amount applied from fertilizers from 1984 to 2013 (B)	Total removed in crop grains from 1984 to 2013 (C)	Final total content in the soil in 2013 (D)	Balance^b^
P content (kg P ha^−1^)					
NTCW	2,322	1,053	120a^c^	2,257a	−988ab
STCW	2,322	1,053	121a	2,105b	−1,149b
FSTCW	2,322	1,053	131a	2,184ab	−1,060ab
FSTW-B/P	2,322	1,053	121a	2,109b	−1,145b
STW-F	2,322	527	80b	2,041c	−728a
Contrast					
NT vs. T	–	–	−6	113**	107
CW vs. W-F	–	–	41*	64*	−421**
CW vs. W-B/P	–	–	10	75*	175*
K content (kg K ha^−1^)					
NTCW	4,490	816	137a	4,363a	−806a
STCW	4,490	816	137a	4,070b	−1,099b
FSTCW	4,490	816	149a	4,223ab	−934ab
FSTW-B/P	4,490	816	138a	4,077b	−1091b
STW-F	4,490	408	91b	3,947b	−860a
Contrast					
NT vs. T	–	–	−6	217*	211*
CW vs. W-F	–	–	46*	93	−239*
CW vs. W-B/P	–	–	11	146	−157

*NTCW* no-till continuous spring wheat, *STCW* spring till continuous spring wheat and *STW-F* spring till spring wheat-fallow. *CW* represents continuous wheat, *NT* no-till, *T* till, *W–B/P* spring wheat–barley/pea and *W-F* spring wheat-fallow.

* and ** Significant at *P* = 0.05 and 0.01, respectively.

^a^Tillage and cropping sequence are *FSTCW* fall and spring till continuous spring wheat, *FSTW–B/P* fall and spring till spring wheat–barley (1994–1999) followed by spring wheat–pea (2000–2013).

^b^P or K balance = Column (C) + column (D) − column (A) − column (B).

^c^Numbers followed by different lowercase letters within a column among treatments in a set are significantly different at *P* ≤ 0.05 by the least square means test.

Reduced amount of P fertilization to crops, P uptake, and/or crop residue returned to the soil probably resulted in lower Olsen-P concentration at 0–7.5 cm in STW-F than the other treatments. Phosphorus fertilizer was applied to spring wheat once in 2 years in STW-F compared to other treatments where fertilization was done annually to spring wheat, barley, and pea. Non-significant differences in P concentration among treatments and depths below 7.5 cm were probably a result of immobile nature of P. It has been well known that P moves slowly relative to N and K in the soil profile (Kuo 1996; Tarkalson et al. 2006). Overall, tillage to a depth of 10 cm had no effect on Olsen-P concentration even after 30 year. Increased crop residue returned to the soil likely increased Olsen-P at 0–7.5 cm, but increased P uptake from subsoil layers probably reduced Olsen-P at 7.5–15 and 15–30 cm under continuous wheat than wheat-fallow and wheat–barley/pea.

As with Olsen-P, the trend in K concentration among treatments and depths was similar (Tables 2, 3). Potassium concentration at 0–7.5 and 7.5–15 cm was lower in STW-F than the other treatments, except for the concentration at 0–7.5 cm in FSTCW. Absence of crops and lack of K fertilization during fallow reduced K concentration in STW-F. At 15–30, 30–60, 60–90, and 90–120 cm, K concentration was not affected by treatments and averaged 153, 100, 96, and 103 mg K kg^−1^, respectively. Similar to Olsen-P concentration, tillage had no effect on K concentration. This was similar to that reported by Lal et al. (1994) and Sainju et al. (2011), but in contrast to that documented by Tarkalson et al. (2006) who found greater K concentration at 0–5 and 5–10 cm in conventional tillage than no-tillage due to increased residue incorporation into the soil. Increased amount of crop residue returned to the soil and/or K fertilization increased K concentration at 0–60 cm under continuous wheat than wheat-fallow. Similar to Olsen-P, K concentration decreased from 0–7.5 to 15–30 cm and then remained constant with depth in all treatments. Application of K fertilizer increased K concentration in surface soil layer, a case similar to that observed for Olsen-P. Significant differences in K concentration among treatments at 7.5–15 cm compared with non-significant differences for P at this layer suggests that K is more mobile than P.

Differences in the rate of P and K fertilizers applied to crops, removal of P and K in crop grains, and soil total P and K contents at 0–120 cm at the end of the experiment resulted in variations in P and K balances among treatments (Table 4). Although soil total P and K contents at 0–120 cm at the end of the experiment were greater in NTCW than STCW, FSTW-B/P, and STW-F, lower P and K fertilization rates to crops and removal in grains resulted in higher P and K balances in STW-F than STCW and FSTW-B/P. Increased P and K fertilization rates compared to grain P and K uptake may have increased soil residual P and K levels, which likely increased P and K losses and therefore negative balances under continuous wheat compared to wheat-fallow. Reduced mineralization of soil organic matter and crop residue likely increased K balance in no-till than conventional till. Both P and K balances were, however, negative in all treatments, suggesting that P and K were lost from the surface soil probably due to surface runoff and leaching after 30 year, a case similar to that reported by various researchers (Kirkby et al. 2011; Wang et al. 2014).

Olsen-P and K concentrations at 0-7.5 cm (25.0–40.0 mg P kg^−1^ and 272–348 mg K kg^−1^, respectively) were greater than the critical levels of 12.0 mg P kg^−1^ and 120 mg K kg^−1^, respectively, for optimum dryland crop production in the northern Great Plains (Agvise Laboratories 2010). As shown above, crop grains were able to remove only 12–22% of applied P and K through fertilizers and annual application of P and K fertilizers can increase P and K losses from the agroecosystem. As a result, P and K fertilization rates can either be reduced or suspended for several years until their concentrations in the soil falls near the critical levels. This will help in reducing the cost of fertilization and improving soil and environmental quality without altering crop yields.

### Soil pH and buffer pH

Soil pH and buffer pH varied among treatments and depths, with a significant treatment × depth interaction (Table 2). Soil pH at 0–7.5 cm was greater in FSTW–B/P and STW-F than STCW and FSTCW (Table 5). At 7.5–15 cm, pH was greater in STW-F than the other treatments, except NTCW. At 15–30, 30–60, 60–90, and 90–120 cm, pH was not different among treatments and averaged 7.65, 8.26, 8.58, and 8.69, respectively. Soil pH was lower under continuous wheat than wheat-fallow at 0–7.5 and 7.5–15 cm and lower than wheat–barley/pea at 0–7.5 cm. Soil pH increased with depth, regardless of treatments.

**Table 5:** Effect of tillage and cropping sequence combination on soil pH, buffer pH, and electrical conductivity (EC) at the 0–120 cm depth in 2013

Tillage and cropping sequence^a^	Soil depth					
	0–7.5 cm	7.5–15 cm	15–30 cm	30–60 cm	60–90 cm	90–120 cm
pH						
NTCW	5.33ab^b^E^c^	6.50abD	7.60C	8.35B	8.58A	8.75A
STCW	5.05bE	6.15bD	7.58C	8.25B	8.63A	8.70A
FSTCW	5.02bE	6.33bD	7.80C	8.30B	8.68AB	8.73A
FSTW-B/P	5.46aE	6.44bD	7.60C	8.15B	8.51A	8.59A
STW-F	5.73aE	7.03aD	7.65C	8.25B	8.50AB	8.66A
Contrast						
NT vs. T	0.29	0.26	−0.09	0.08	−0.08	0.04
CW vs. W-F	−0.68***	−0.88**	−0.08	0.01	0.13	0.04
CW vs. W-B/P	−0.43*	−0.11	0.20	0.15	0.16	0.14
Buffer pH						
NTCW	6.45bE	7.10abD	7.43C	7.60B	7.70AB	7.73A
STCW	6.38bE	7.00bD	7.43C	7.58B	7.68A	7.70A
FSTCW	6.43bE	7.05bD	7.45C	7.60B	7.70AB	7.73A
FSTW-B/P	6.66aD	7.13abC	7.44B	7.58B	7.69AB	7.70A
STW-F	6.80aE	7.24aD	7.44C	7.59B	7.66AB	7.72A
Contrast						
NT vs. T	0.05	0.08	−0.01	0.01	0.01	0.01
CW vs. W-F	−0.43***	−0.24**	−0.01	−0.01	0.01	−0.01
CW vs. W-B/P	−0.24*	−0.08	−0.01	0.03	0.01	0.03
EC (dS m^−1^)						
NTCW	0.17B	0.10B	0.16B	0.25A	0.28A	0.30bA
STCW	0.18BC	0.16C	0.22AB	0.24AB	0.28A	0.28bA
FSTCW	0.19C	0.20C	0.28B	0.29B	0.38AB	0.47aA
FSTW-B/P	0.23B	0.21B	0.30AB	0.36AB	0.43A	0.45aA
STW-F	0.19B	0.20B	0.26AB	0.31A	0.34A	0.33bA
Contrast						
NT vs. T	−0.02	−0.06	−0.09	−0.03	−0.05	−0.07*
CW vs. W-F	−0.01	−0.04	−0.04	−0.07	−0.06	−0.05
CW vs. W-B/P	−0.04	−0.01	−0.02	−0.07	−0.05	−0.02

*NTCW* no-till continuous spring wheat, *STCW* spring till continuous spring wheat and *STW-F* spring till spring wheat-fallow. *CW* represents continuous wheat, *NT* no-till, *T* till, *W–B/P* spring wheat–barley/pea and *W-F* spring wheat-fallow.

*, **, and *** Significant at *P* = 0.05, 0.01, and 0.001, respectively.

^a^Tillage and cropping sequence are *FSTCW* fall and spring till continuous spring wheat, *FSTW–B/P* fall and spring till spring wheat–barley (1994–1999) followed by spring wheat–pea (2000–2013).

^b^Numbers followed by different lowercase letters within a column among treatments in a set are significantly different at *P* ≤ 0.05 by the least square means test.

^c^Numbers followed by different uppercase letters within a row among soil depths in a set are significantly different at *P* ≤ 0.05 by the least square means test.

The trend for soil buffer pH among treatments was similar to pH (Table 5). At 0–7.5 cm, buffer pH was greater in FSTW–B/P and STW-F than the other treatments. At 7.5–15 cm, buffer pH was greater in STW-F than STCW and FSTCW. At other depths, buffer pH was not different among treatments and averaged 7.44, 7.59, 7.69, and 7.72 at 15–30, 30–60, 60–90, and 90–120 cm, respectively. Buffer pH was lower under continuous wheat than wheat-fallow at 0–7.5 and 7.5–15 cm and lower than wheat–barley/pea at 0–7.5 cm. Buffer pH was 1.07–1.41 units greater at 0–7.5 cm and 0.21–0.85 units greater at 7.5–15.0 cm than pH. At other depths, buffer pH was either similar to or less than pH.

The greater soil pH and buffer pH at 0–7.5 and 7.5–15 cm in FSTW–B/P and STW-F were probably a result of reduced amount of N fertilizer applied. Nitrogen fertilizer was either applied at 5 kg N ha^−1^ to pea compared with 34–70 kg N ha^−1^ applied to spring wheat and barley in FSTW–B/P in each year or was not applied during the fallow phase in STW-F. In contrast, N fertilizer was applied to spring wheat at 34–70 kg N ha^−1^ every year in NTCW, STCW, and FSTCW. Continuous application of NH_4_-based N fertilizers to crops can reduce soil pH, resulting in the development of infertile soils and decreased crop yields (Liebig et al. 2002; Tumuslime et al. 2011; Schroder et al. 2011). Several researchers (Lal et al. 1994; Liebig et al. 2002) have found that soil pH was higher in crop rotations containing legumes and nonlegumes than continuous nonlegumes, a case similar to that obtained for higher pH and buffer pH in FSTW–B/P than FSTCW in our experiment (Table 4). Tillage had no effect on soil pH and buffer pH. This was similar to that observed by Lal et al. (1994), but different from that found by Tarkalson et al. (2006) who reported that soil pH varied with tillage at various depths due to variations in depth of incorporation of N fertilizer into the soil. Greater differences in buffer pH and pH at 0-7.5 cm among treatments showed that the acidity in the surface soil layer can be reduced by liming, especially in NTCW, STCW, and FSTCW. Because soil pH was >6.0 below 7.5 cm, lime can be applied at variables rates depending on soil pH among treatments in the surface layer without the need for incorporating it into the soil to neutralize acidity and increase the availability of most nutrients, thereby improving crop yields.

### Soil calcium and magnesium

Soil Ca and Mg concentrations varied among depths, with a significant treatment × depth interaction (Table 2). Soil Ca concentration at 0–7.5 cm was greater in FSTW–B/P and STW-F than NTCW, STCW, and FSTCW (Table 6). At 7.5–15, 15–30, 30–60, 60–90, and 90–120 cm, Ca concentration was not different among treatments and averaged 1.88, 3.65, 4.68, 4.58, and 4.19 g Ca kg^−1^, respectively. At 0-7.5 cm, Ca concentration was lower under continuous wheat than wheat-fallow and wheat–barley/pea. Calcium concentration increased with depth from 0–7.5 to 30–60 cm and then remained constant thereafter in all treatments, except for FSTCW.

**Table 6:** Effect of tillage and cropping sequence combination on soil Ca, Mg, and Na concentrations at the 0–120 cm depth in 2013

Tillage and cropping sequence^a^	Soil depth					
	0–7.5 cm	7.5–15 cm	15–30 cm	30–60 cm	60–90 cm	90–120 cm
Ca concentration (g Ca kg^−1^)						
NTCW	0.99b^b^C^c^	1.60C	3.55B	5.32A	4.67A	3.98B
STCW	0.89bC	1.61C	2.97B	4.65A	4.72A	4.31A
FSTCW	0.99bC	1.78C	4.09B	5.34A	4.51AB	4.05B
FSTW-B/P	1.26aB	2.06B	3.76A	4.66A	4.32A	4.30A
STW-F	1.29aB	2.36B	3.89A	4.67A	4.68A	4.25A
Contrast						
NT vs. T	0.04	−0.09	0.02	0.32	0.05	−0.20
CW vs. W-F	−0.40**	−0.75	−0.92	−0.02	0.04	0.06
CW vs. W-B/P	−0.27*	−0.28	0.33	0.68	0.19	−0.25
Mg concentration (g Mg kg^−1^)						
NTCW	0.21E	0.34bDE	0.48D	0.70C	1.23B	1.52A
STCW	0.19E	0.35bDE	0.47CD	0.65C	1.19B	1.36A
FSTCW	0.21D	0.40bCD	0.50C	0.83B	1.34A	1.53A
FSTW-B/P	0.25D	0.38abD	0.50CD	0.75C	1.18B	1.40A
STW-F	0.25D	0.43aCD	0.54BC	0.73B	1.13A	1.40A
Contrast						
NT vs. T	0.01	−0.04	0.01	−0.04	−0.04	0.08
CW vs. W-F	−0.06*	−0.08*	0.07	−0.08	0.06	−0.04
CW vs. W-B/P	−0.04	0.02	0.00	0.08	0.16	0.13
Na concentration (mg Na kg^−1^)						
NTCW	14.5ab^b^B	15.5B	16.0B	19.0B	30.3bB	58.8bA
STCW	14.8bB	14.3B	15.3B	19.0B	36.8bB	65.5bA
FSTCW	15.8abC	15.3C	18.5C	24.8C	66.8aB	119.3aA
FSTW-B/P	16.6aC	21.0C	30.4C	37.4C	64.5aB	102.6aA
STW-F	12.4bB	14.4B	16.9B	19.5B	27.0bB	57.4bA
Contrast						
NT vs. T	−0.8	−0.7	−0.9	−9	−21.5*	−33.6*
CW vs. W-F	2.4	−0.1	−1.6	−0.5	9.8	8.1
CW vs. W–B/P	−0.8	−5.7	−11.9	−12.6	2.3	16.7

*NTCW* no-till continuous spring wheat, *STCW* spring till continuous spring wheat and *STW-F* spring till spring wheat-fallow. *CW* represents continuous wheat, *NT* no-till, *T* till, *W–B/P* spring wheat–barley/pea and *W-F* spring wheat-fallow.

* and ** Significant at *P* = 0.05 and 0.01, respectively.

^a^Tillage and cropping sequence are *FSTCW* fall and spring till continuous spring wheat, *FSTW–B/P* fall and spring till spring wheat–barley (1994–1999) followed by spring wheat–pea (2000–2013).

^b^Numbers followed by different lowercase letters within a column among treatments in a set are significantly different at *P* ≤ 0.05 by the least square means test.

^c^Numbers followed by different uppercase letters within a row among soil depths in a set are significantly different at *P* ≤ 0.05 by the least square means test.

The trend for soil Mg concentration was similar to Ca concentration. At 7.5–15 cm, Mg concentration was greater in STW-F than NTCW, STCW, and FSTCW (Table 6). At 0–7.5, 15–30, 30–60, 60–90, and 90–120 cm, Mg concentration was not different among treatments and averaged 0.22, 0.50, 0.73, 1.21, and 1.44 g Mg kg^−1^, respectively. Magnesium concentration was lower under continuous wheat than wheat-fallow at 0–7.5 and 7.5–15 cm. Unlike Ca concentration, Mg concentration increased with depth in all treatments.

Greater Ca and Mg concentrations at 0–7.5 and 7.5–15 cm in STW-F and FSTW–B/P than the other treatments were similar to higher soil pH and buffer pH in these treatments (Table 5). It is likely that the absence of N fertilization to crops during fallow or reduced N fertilization to pea increased soil pH and therefore Ca and Mg concentrations in STW-F and FSTW–B/P compared with the other treatments. In contrast, increased soil acidity resulting from N fertilization to spring wheat every year probably increased dissolutions of Ca and Mg which were either taken up by the crop or moved down the soil profile, resulting in lower Ca and Mg concentrations at the surface layer and increased with depth in NTCW, STCW, and FSTCW. Increased Mg concentration with depth as opposed to similar levels of Ca concentration below 30 cm indicates that the proportion of Mg-containing minerals increased with depth while the amount of Ca-containing minerals remained the same. As with pH and buffer pH, tillage had no effect on these nutrients, a case in contrast to those reported for various levels of Ca and Mg in no-tillage and conventional tillage systems at various depths (Tarkalson et al. 2006). This could be a result of differences in the amount of crop residue returned to the soil among tillage systems. Mean annualized crop residue returned to the soil was not different among NTCW, STCW, and FSTCW in this study, but was greater in no-till than conventional till in the experiment described by Tarkalson et al. (2006). Increased amount of crop residue returned to the soil can increase soil Ca and Mg concentrations (Lal et al. 1994; Liebig et al. 2002).

### Soil sodium, sulfate-sulfur, and zinc

Soil Na and SO_4_–S concentrations varied among treatments and depths and Zn concentration among depths (Table 2). The treatment × depth interaction was significant for Na, SO_4_–S, and Zn concentrations. At 0–7.5 cm, Na concentration was greater in FSTW–B/P than STCW and STW-F (Table 6). At 60–90 and 90–120 cm, Na concentration was greater in FSTCW and FSTW–B/P than NTCW, STCW, and STW-F. At 7.5–15, 15–30, and 30–60 cm, Na concentration was not different among treatments and averaged 16.1, 19.4, and 25.9 mg Na kg^−1^, respectively. At 60–90 and 90–120 cm, Na concentration was lower in no-till than conventional till. Similar to Mg concentration, Na concentration increased with depth.

Increased tillage intensity and/or amount of crop residue returned to the soil probably increased Na concentration at 0–7.5, 60–90, and 90–120 cm in FSTCW and FSTW–B/P compared with other treatments. It is likely that increased mineralization of crop residue and soil organic matter due to enhanced tillage increased mobility of Na, some of which moved down the soil profile, thereby increasing Na concentration in FSTCW and FSTW-B/P, especially at deeper layers. This was similar to that reported by Sainju et al. (2011) who found greater soil Na concentration in conventional tillage than no-tillage, where tillage was conducted to a depth of 20 cm compared to 10 cm in our study. The increased Na concentration with depth was proportional to increased soil pH and Ca and Mg concentrations, suggesting that continuous N fertilization to crops increased dissolution of Na that was either taken by the crop or moved down from the surface to the subsurface layers.

Soil SO_4_–S concentration at 90–120 cm was greater in FSTCW and FSTW–B/P than NTCW, STCW, and STW-F (Table 3). At 0–7.5, 7.5–50, 15–30, 30-–60, and 60–90 cm, SO_4_–S concentration was not different among treatments and averaged 8.0, 5.9, 5.7, 6.7, 10.4 mg SO_4_–S kg^−1^, respectively. At 90–120 cm, SO_4_–S concentration was lower in no-till than conventional till. Similarly to Na concentration, enhanced tillage and/or increased amount of crop residue returned to the soil likely increased SO_4_–S concentration at 90–120 cm in FSTCW and FSTW-B/P. Although not significant, SO_4_–S concentration was lower under continuous wheat than wheat-fallow and wheat–barley/pea. This was similar to that observed by Sainju et al. (2011) who reported greater SO_4_–S concentration at subsurface layers in wheat-fallow than continuous wheat. Increased SO_4_–S concentration with depth in FSTCW and FSTW–B/P as opposed to similar levels at all depths in the other treatments suggest that enhanced tillage intensity increased mineralization of crop residue and soil organic matter that accelerated the mobility of SO_4_–S, some of which moved down the soil profile and accumulated in the deeper layers.

In contrast to Na and SO_4_–S concentrations, Zn concentration at 0–7.5 cm was greater in NTCW than FSTW–B/P and STW-F (Table 7). At 7.5–15, 15–30, 30–60, 60–90, and 90–120 cm, Zn concentration was not different among treatments and averaged 0.45, 0.20, 0.20, 0.21, and 0.29 mg Zn kg^−1^, respectively. Zinc concentration was greater under continuous wheat than wheat-fallow at 0–7.5 cm and greater than wheat–barley/pea at 90–120 cm. Reduced soil disturbance and greater amount of crop residue returned to the soil appeared to increase Zn concentration at 0–7.5 cm in NTCW. Increased N fertilization appeared to increase Zn concentration under continuous wheat than wheat-fallow and wheat–barley/pea. As with P and K concentrations, Zn concentration decreased from 0–7.5 to 7.5–15 cm and remained constant with depth thereafter.

**Table 7:** Effect of tillage and cropping sequence combination on soil Zn concentration and cation exchange capacity at the 0–120 cm depth in 2013

Tillage and cropping sequence^a^	Soil depth					
	0–7.5 cm	7.5–15 cm	15–30 cm	30–0 cm	60–90 cm	90–120 cm
Zn concentration (mg Zn kg^−1^)						
NTCW	2.33a^b^A^c^	0.42B	0.14B	0.16B	0.14B	0.21B
STCW	1.88abA	0.40B	0.21B	0.17B	0.17B	0.36B
FSTCW	1.71abA	0.52B	0.19B	0.21B	0.23B	0.38B
FSTW-B/P	1.15bA	0.56B	0.25B	0.21B	0.18B	0.20B
STW-F	0.96bA	0.35B	0.20B	0.24B	0.32B	0.30B
Contrast						
NT vs. T	0.54	−0.04	−0.06	−0.03	−0.06	−0.16
CW vs. W-F	0.92*	0.05	0.01	−0.07	−0.15	0.06
CW vs. W-B/P	0.56	−0.04	−0.06	−0.00	0.05	0.18*
CEC (cmol_c_ kg^−1^)						
NTCW	14.3aC	11.6C	22.2B	32.8A	33.9A	33.1A
STCW	14.5aC	12.9C	19.3B	29.0A	34.0A	33.5A
FSTCW	14.6aC	13.3C	25.1B	34.0A	34.2A	33.8A
FSTW-B/P	13.4aC	14.2C	23.5B	30.0A	32.0A	33.9A
STW-F	11.9bC	15.9BC	24.3AB	29.7A	33.2A	33.3A
Contrast						
NT vs. T	−0.3	−1.5	0.0	1.3	−0.2	−0.6
CW vs. W-F	2.6**	−3.0	−5.0	−0.7	0.8	0.2
CW vs. W–B/P	1.2	−0.9	1.6	4.0	2.2	−0.1

*NTCW* no-till continuous spring wheat, *STCW* spring till continuous spring wheat and *STW-F* spring till spring wheat-fallow. *CW* represents continuous wheat, *NT* no-till, *T* till, *W–B/P* spring wheat–barley/pea and *W-F* spring wheat-fallow.

* and ** Significant at *P* = 0.05 and 0.01, respectively.

^a^Tillage and cropping sequence are *FSTCW* fall and spring till continuous spring wheat, *FSTW–B/P* fall and spring till spring wheat–barley (1994–1999) followed by spring wheat–pea (2000–2013).

^b^Numbers followed by different lowercase letters within a column among treatments in a set are significantly different at *P* ≤ 0.05 by the least square means test.

^c^Numbers followed by different uppercase letters within a row among soil depths in a set are significantly different at *P* ≤ 0.05 by the least square means test.

### Soil cation exchange capacity and electrical conductivity

Soil CEC varied among treatments and depths and EC among depths, with a significant treatment × depth interaction for both CEC and EC (Table 2). Soil CEC at 0–7.5 cm was lower in STW-F than NTCW, STCW, FSTCW, and FSTW–B/P (Table 7). At 7.5–15, 15–30, 30–60, 60–90, and 90–120 cm, CEC was not different among treatments and averaged 13.6, 22.9, 31.1, 33.5, and 33.5 cmol_c_ kg^−1^, respectively. The CEC was greater under continuous wheat than wheat-fallow at 0–7.5 cm. The CEC increased from 7.5–15 to 30–60 cm and remained constant with depth for all treatments.

Reduced amount of crop residue returned to the soil due to the absence of crops during fallow likely decreased CEC at the surface layer in STW-F compared with the other treatments. Sainju et al. (2011) also found lower CEC in wheat-fallow than continuous wheat after 9 years in dryland cropping systems in western Montana. As with most other soil parameters, tillage had no effect on CEC. This was in contrast to those reported by several researchers (Lal et al. 1994; Tarkalson et al. 2006) who found that CEC was greater in no-tillage than conventional tillage at the surface soil. Differences among tillage depths and the amount of crop residue returned to the soil likely resulted in variation in CEC among tillage systems in various locations. Increased CEC below 15 cm was due to increased Ca, Mg, and Na concentrations (Table 6).

In contrast to CEC, EC at 90–120 cm was greater in FSTCW and FSTW–B/P than NTCW, STCW, and STW-F (Table 5). At 0–7.5, 7.5–15, 15–30, 30–60, and 60–90 cm, EC was not different among treatments and averaged 0.19, 0.17, 0.24, 0.29, and 0.34 dS m^−1^, respectively. The EC was lower in no-till than conventional till at 90–120 cm. Increased tillage intensity and/or amount of crop residue returned to the soil likely increased EC at 90–120 cm in FSTCW and FSTW-B/P. Greater EC with these treatments at this depth were also associated with higher Na and SO_4_–S concentrations (Tables 3, 6), suggesting that increased accumulation of these nutrients increased soil salinity at deeper soil layer. The EC <0.25 dS m^−1^ at 0–7.5 and 7.5–15 cm indicates that soils are not saline at the surface layers and are optimal for crop growth and microbial activity (Liebig et al., 2002). Our results were similar to that reported by Sainju et al. (2011) who found greater EC with conventional tillage than no-tillage at 30–60 cm, but in contrast to higher EC in wheat-fallow than continuous wheat due to increased Ca, Mg, and Na concentrations. Similar to CEC, EC also increased with depth below 15 cm, suggesting increased salinity.

### Implication of management practices

Mean annualized crop yield was not different among NTCW, STCW, FSTCW, and FSTW-B/P, and the yield was greater in these treatments than STW-F (Figure 1). Most soil nutrients and chemical properties at the surface soil in FSTW–B/P were either greater than or similar to the other treatments. As a result, FSTW–B/P can be used as a superior management practice to reduce N fertilization rate and maintain long-term soil fertility and crop yield. Tillage had no effect on dryland wheat and pea yields (Lenssen et al. 2007, 2014) and also on annualized crop yield and soil properties as observed in this experiment. Furthermore, crop rotation had little effect on annualized crop yield and soil properties. As a result, no-tillage with legume-nonlegume crop rotation may be used to enhance the long-term sustainability of dryland soil fertility and crop yields with reduced chemical fertilizer and tillage-related inputs. Because of reduced crop yields, lower nutrient concentrations, and degraded chemical properties, conventional tillage with crop-fallow system should be avoided in dryland cropping systems.

## Conclusions

Annualized crop yield, soil nutrients, and chemical properties varied among treatments due to variations in tillage intensity and cropping sequences after 30 years. At the surface layer, soil Olsen-P, K, Zn, and Na concentrations and CEC were greater, but pH, buffer pH, and Ca concentration were lower in NTCW, STCW, and FSTCW than STW-F. At the subsurface layers, EC, Na, and SO_4_–S concentrations were greater in FSTW–B/P and FSTCW than the other treatments. Olsen-P, K, and Zn concentrations decreased, but Ca, Mg, Na, and SO_4_–S concentrations, pH, buffer pH, EC, and CEC increased with soil depth. Annualized crop yield was lower in STW-F than the other treatments. Long-term reduced tillage with continuous cropping increased P, K, and Zn concentrations and CEC by reducing soil disturbance and increasing crop residue returned to the soil and annualized crop yield, but reduced pH and basic cations at the surface layer due to increased N fertilizer application compared with the traditional system of conventional tillage with spring wheat-fallow. Results suggest that no-tillage with legume-nonlegume crop rotation may be used as a viable management practice to sustain the long-term dryland soil fertility and crop yields with reduced chemical and energy inputs. Because of increased residual soil P and K concentrations and their losses from agroecosystem, P and K fertilization rates can either be reduced or suspended for several years until their concentrations falls near the critical levels.

## Abbreviations

CEC: cation exchange capacity
EC: electrical conductivity
FSTCW: fall and spring till continuous spring wheat
FSTW-B/P: fall and spring till spring wheat–barley (1984–1999) followed by spring wheat–pea (2000–2013)
NTCW: no-till continuous spring wheat
STCW: spring till continuous spring wheat
STW-F: spring till spring wheat-fallow
